# A Flexible Microcontroller-Based Data Acquisition Device

**DOI:** 10.3390/s140609755

**Published:** 2014-06-02

**Authors:** Darko Hercog, Bojan Gergič

**Affiliations:** Institute of Automation, Faculty of Electrical Engineering and Computer Science, University of Maribor, Smetanova ulica 17, Maribor 2000, Slovenia; E-Mail: bojan.gergic@um.si

**Keywords:** data acquisition, DAQ, microcontroller, analogue-to-digital converter, ADC, USB, HID, LabVIEW, GUI, data logging

## Abstract

This paper presents a low-cost microcontroller-based data acquisition device. The key component of the presented solution is a configurable microcontroller-based device with an integrated USB transceiver and a 12-bit analogue-to-digital converter (ADC). The presented embedded DAQ device contains a preloaded program (firmware) that enables easy acquisition and generation of analogue and digital signals and data transfer between the device and the application running on a PC via USB bus. This device has been developed as a USB human interface device (HID). This USB class is natively supported by most of the operating systems and therefore any installation of additional USB drivers is unnecessary. The input/output peripheral of the presented device is not static but rather flexible, and could be easily configured to customised needs without changing the firmware. When using the developed configuration utility, a majority of chip pins can be configured as analogue input, digital input/output, PWM output or one of the SPI lines. In addition, LabVIEW drivers have been developed for this device. When using the developed drivers, data acquisition and signal processing algorithms as well as graphical user interface (GUI), can easily be developed using a well-known, industry proven, block oriented LabVIEW programming environment.

## 1. Introduction

Data acquisition (DAQ) is the process of measuring an electrical or physical phenomenon using a computer. The complete DAQ system consists of sensors, DAQ hardware, and a computer with programmable software. The sensor performs a conversion of the physical phenomenon into an electrical signal. This signal is further converted into digital numeric values by DAQ hardware, which is controlled by a software program developed using various general purpose programming languages (C, LabVIEW, Visual Basic, MATLAB). In addition to the hardware control, such DAQ software usually also includes data analysis, data visualisation, and data storage algorithms. Commercially available DAQ devices range from relatively simple devices up to very sophisticated ones. The latter ones contain ADC, digital-to-analogue converters (DAC), digital inputs/outputs, counters, computer bus, circuits for digital routing and clock generation, circuits for automatic offset and gain calibrations, *etc.* Such large-scale DAQ devices are very powerful and suitable for applications where functionality has precedence over price, such as within the research area and industrial applications. There are, however, also several applications where such state-of-the-art solutions are sometimes unnecessary. In the education process or simple low cost applications, for example, less efficient, scalable, and low cost DAQ devices are welcome. Most of DAQ hardware producers also provide low-cost DAQ solutions but such devices cost at least 150€ and that is still too expensive for low budget applications. Nowadays, very simple and low cost DAQ devices could be realised using microcontrollers with integrated analogue-to-digital converters (ADC). A microcontroller is a small computer on a single integrated circuit containing a processing unit, memory, and programmable input/output peripherals.

Several microcontroller-based data acquisition (DAQ) systems have been presented by different authors. In the majority of the presented solutions the developed DAQ devices are not general purpose DAQs, but rather have been specially designed for specific DAQ applications, such as temperature or humidity measurements. In [1] the authors present a microcontroller-based DAQ that is applied to decentralised renewable energy plants whilst in [2], for thermoelectric property measurements. In the latter case the DAQ is based on a PIC18F4550 microcontroller. The resolution of the microcontroller’s internal ADC (10-bit) has been improved by using external 22-bit delta-sigma ADC with a performance of 60 samples per second (S/s). The DAQ GUI was developed in Delphi. In [3] a data acquisition and control system for high-speed gamma-ray tomography based on the USB and Ethernet communication protocols has been designed. This system is based on Microchip’s PIC18F4550 and PIC18F4620 whilst the DAQ software is realised using LabVIEW. In [4] the authors present a microcontroller-based USB DAQ device for radiation detection and environmental monitoring purposes. The developed DAQ, which is based on an 8-bit AVR microcontroller, has 8 analogue input channels, 10-bit ADC and enables acquisition of analogue input with a maximum sampling rate of 50 kS/s. In [5] the authors present a low-cost USB DAQ based on the PIC18C442 microcontroller, with integrated 10-bit ADC. 12-bit A/D and D/A resolutions have been achieved using external A/D (TI ADS803) and D/A (TI DAC7545) converters. A USB data acquisition system for humidity and temperature measurements is presented in [6]. This DAQ device is based on PIC18F4550 and enables acquisition of 8 analogue inputs with 10-bit resolution. Humidity measurement is achieved using an HIH 4000 humidity sensor whilst for the temperature using an LM35 temperature sensor. An application program on the PC was developed using Visual Basic. Datta *et al.* [7] presented a DAQ device for the current, voltage, frequency, and power factor measurements of electric motors. The DAQ is connected to the PC using RS-485 serial interface, and enables sampling rates of up to 960 S/s. An application program on the PC was developed using Visual Basic. In [8] Gupta *et al.* describe a low-cost DAQ for electrocardiogram (ECG) monitoring. The sampling rate of the developed DAQ is 1 kS/s and is connected to a PC using the RS-232 serial bus, whilst an application program was developed using MATLAB. In [9] the author presents a microcontroller-based DAQ for differential thermal analysis. The DAQ is based on the PIC18F4550 microcontroller and two K-type thermocouples. Cold-junction compensation and digitisation of a signal from a type-K thermocouple was achieved using a Maxim MAX6675 chip connected to the microcontroller via an SPI bus. The DAQ enables sampling rates of up to 4 S/s. The user interface was realised using LabVIEW. A similar project is presented in [10]. In this case the device is based on a PIC18F4550 microcontroller and an AD595AQ monolithic thermocouple amplifier that enables temperature measurements of K type thermocouples. The GUI was developed in Visual Basic. In [11] the authors present a USB air quality monitoring system that can measure and analyse the concentrations of major air pollutant gases. In [12] the same authors presented a modified solution for an indoor environmental monitoring system that is based on the IEEE 1451 standard. In both cases, the developed device was based on PIC18F4550, whilst the communication and the GUI were realised using LabVIEW. In [13] a microcontroller based data acquisition system is presented for slowly varying signals. This DAQ is based on PIC12F675 and enables acquisition of four analogue inputs with 10-bit resolution. DAQ is connected to a PC using a serial RS-232 bus. An application program was developed using Visual Basic and MSComm components. Over recent times usage of the popular Arduino platform has been increasing in DAQ applications. The Arduino Uno development board, which is based on an 8-bit Atmel microcontroller, has been successfully used in many applications such as low-cost platforms for studying lymphatic biomechanics *in vitro* [14], a LED stimulator for vision research [15], an open source colorimeter [16], an educational system for teaching the fundamentals of photovoltaic cells [17], electrochemical etching [18], and others. In addition to the stated references, there are also some off-the-shelf USB DAQ devices available on the market. The key features of those devices that are comparable with the presented solution (USB interface, 12-bit ADC) are summarised in Table 1.

This paper presents a flexible, custom-made, microcontroller-based data acquisition device. The key component of the presented device, named as SimpleDAQ, is a microcontroller with an integrated USB transceiver and 12-bit ADC. This SimpleDAQ has been developed as a USB human interface device (HID) because most of operating systems natively support this type of USB class and consequently any installation of additional USB drivers is unnecessary. The SimpleDAQ contains a preloaded program (firmware) that enables easy acquisition and the generation of analogue, digital signals, and data transfer between the device and the application running on a PC via USB. Unlike many of the above-mentioned solutions, where the microcontroller peripheral is fixed, in the presented solution the peripheral is flexible and can be easily configured to customised needs without reprogramming the microcontroller. When using the developed configuration utility, a majority of device pins can be configured as analogue input, digital input/output, PWM output or one of serial peripheral interface (SPI) lines. In this way the device can be easily tailored to the needs of a final DAQ application. In addition, LabVIEW drivers have been developed for the presented device. LabVIEW is a well-known, industry-proven, block programming environment, commonly used for data acquisition, instrument control, and industrial automation on a variety of platforms (Microsoft Windows, Linux, and Mac). When using the developed drives, data acquisition and data analysis algorithms as well as graphical user interface (GUI), can be easily developed using LabVIEW.

The remainder of this paper is organised as follows: Section 2 contains a short description of the developed device’s hardware. In Section 3 the firmware, configuration utility, and LabVIEW driver for the developed device are briefly described. Section 4 contains a short description of those applications that are based on the presented platform. Finally, the conclusions are stated in Section 5.

## 2. DAQ Hardware Overview

This section contains a short hardware overview of developed DAQ device. The section begins with microcontroller selection requirements and continues with a short description of the selected microcontroller and developed DAQ hardware.

### 2.1. Microcontroller Selection

Selection of an appropriate microcontroller for our DAQ device was taken according to the following four main initial requirements:

1. Microcontroller must have integrated USB 2.0 stack. The data exchange between a PC and the microcontroller must be realised directly using universal serial bus (USB) without any additional components such as USB to serial converters. In this way, the device would be simple and as low-cost as possible.
2. Microcontroller must have integrated ADC with at least 12-bit resolution. This resolution should be good enough for a majority of data acquisition applications.
3. Microcontroller must be procurable as a dual in-line package (DIP or DIL) and also in a surface mount device (SMD) package. The DIP package is very useful for experimentation because it can be plugged directly into an experimental board (breadboard).
4. Microcontroller must be low-cost. The main idea was to develop a low-cost DAQ device therefore the limit was set at 5€ per unit.

In regard to the stated requirements and detailed market review, a Microchip PIC18F27J53 microcontroller was selected as our main platform. This microcontroller is a member of the Microchip PIC18F47J53 [24] family of microcontrollers that incorporates a fully-featured USB communications module with a built-in transceiver that is compliant with the USB Specification Revision 2.0. The microcontroller also includes two independent Enhanced USARTs and two Master Synchronous Serial Port (MSSP) modules, capable of both Serial Peripheral Interface (SPI) and I2C (Master and Slave) modes of operation. This family of microcontrollers is ideal for applications requiring cost-effective, low-power USB applications with more code space and peripheral flexibility set within a small package. The key features of the selected PIC18F27J53 microcontroller are summarised in Table 2.

### 2.2. SimpleDAQ Chip

**Figure 1.**
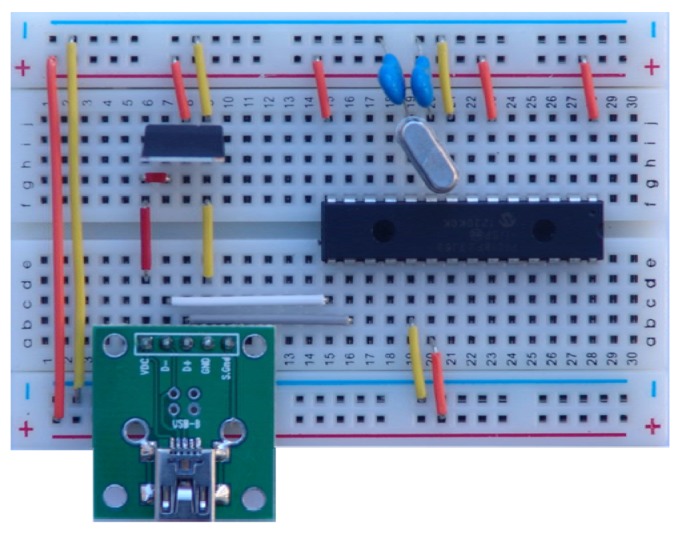
Example of using the SimpleDAQ chip on the breadboard.

The SimpleDAQ chip is actually a Microchip PIC18F27J53 microcontroller [24] with preloaded, custom-developed firmware (the firmware is described in more detail in Section 3.1). The deployed firmware enables the acquisition and generation of analogue/digital signals and data transfer between the chip and the computer using USB serial bus. The SimpleDAQ chip includes a built-in USB transceiver, 12-bit A/D converter, and 18 flexible I/O pins. These pins could be configured as analogue inputs, digital inputs/outputs, PWM outputs, or one of three SPI (Serial Peripheral Interface Bus) lines. The SimpleDAQ chip is procurable within a 28-pin SPDIP package that can be easily plugged directly into the breadboard (Figure 1).

For the basic operation of the SimpleDAQ chip on the breadboard, only a few additional external components are necessary *i.e.*, oscillator with capacitors, USB connector, and 3.3 V power supply (Figure 1). Using 5 V to 3.3 V voltage regulators the power can be obtain directly from the USB bus, as shown in Figure 1.

The resolution of the internal ADC is 12-bits, and the input range is from 0 V to 3.3 V. The code width of the SimpleDAQ chip is as follows:

(1) $$\text{code width}=\frac{\text{input range}}{2^{\text{resolution in bits}}}=\frac{3.3}{2^{12}}=805.7\mu \text{V}$$

### 2.3. SimpleDAQ Module

**Figure 2.**
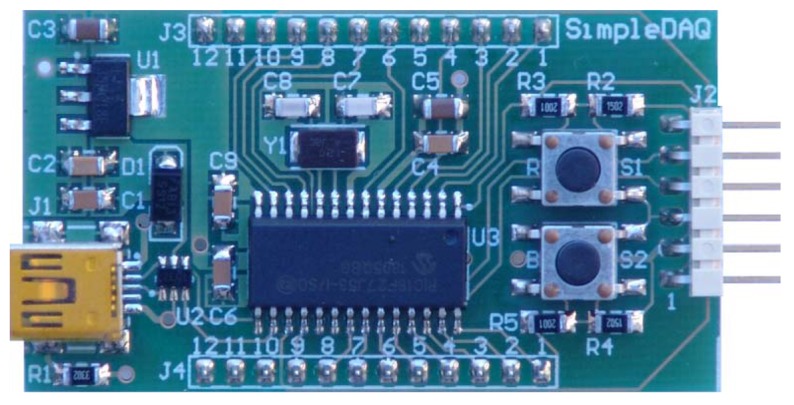
SimpleDAQ module.

On the basis of the SimpleDAQ chip, a SimpleDAQ module has been further developed. This module contains all the components (oscillator, USB connector, *etc.*) that are necessary for the basic operation of the SimpleDAQ chip. This module is powered from USB; therefore any external power is unrequired. The module contains a SimpleDAQ chip (labelled U3 in Figure 2) within the SMD package, an oscillator (Y1), mini USB connector (J1), voltage regulator (U1) for power supply from USB, reset button (R,S1), general purpose button (B,S2), two 12 pin extension connectors (J3 and J4) for the customary application board, debugger connector (J2) and other less necessary components. The module can be plugged directly into the breadboard, or it can be used in combination with a custom-developed application board.

## 3. DAQ Software Overview

The SimpleDAQ software includes the following parts: (1) the firmware that is deployed on the microcontroller, (2) SimpleDAQ configuration utility that enables customisation of microcontroller’s peripheral to the customised needs, and (3) a LabVIEW driver that enables easy creation of data acquisition/generation, data analysis, and data storage algorithms using a LabVIEW development environment.

### 3.1. The Firmware

SimpleDAQ firmware has been developed using Microchip MPLAB® X Integrated Development Environment (IDE). MPLAB X IDE is a free software program that runs on a PC (Windows, Mac OS, Linux) and enables the development of embedded applications on Microchip’s PIC microcontrollers. The firmware has been written in ANSI C language and compiled into the binary code using an MPLAB C Compiler for the PIC18 family of 8-bit MCUs (MPLAB C18).

**Figure 3.**
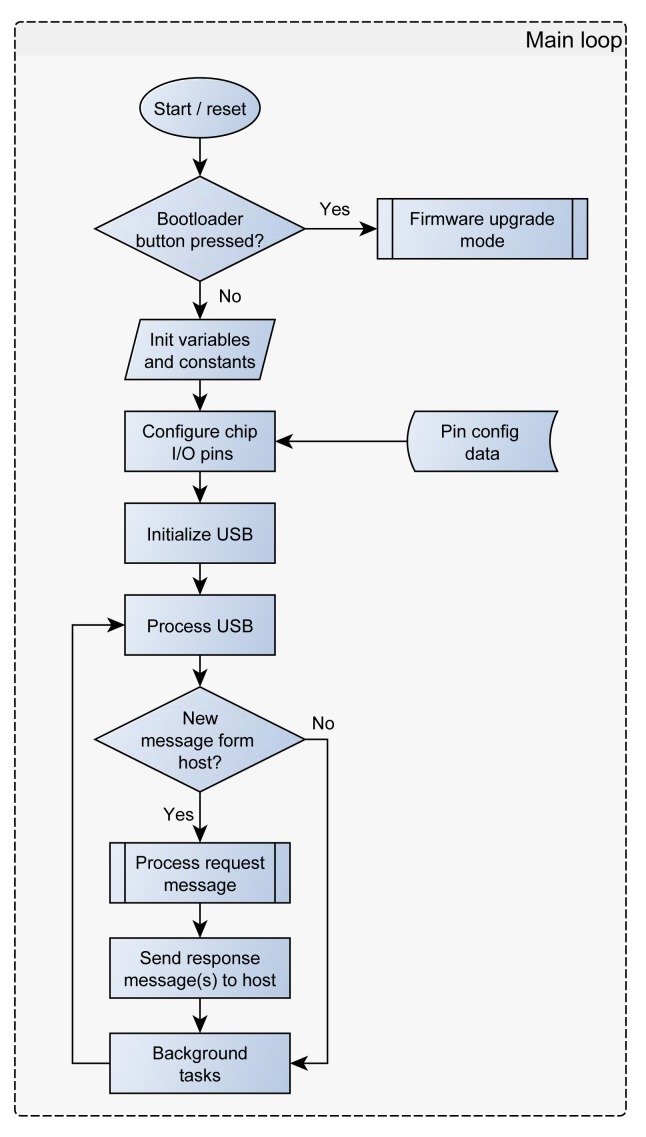
SimpleDAQ firmware flowchart: Main loop.

SimpleDAQ is developed as a USB human interface device (HID). The USB HID class is a part of the USB specification for computer peripherals: it specifies a device class for human interface devices such as keyboards, mice, game controllers, and other devices [25]. Most of operating systems natively support the USB HID class, therefore the end-users do not need to install additional drivers. The maximum transfer rate for USB HID devices is limited to 64 kB/s. SimpleDAQ firmware is developed in the form of a simple server application *i.e.*, the firmware periodically checks for incoming requests (messages) from USB (Figure 3).

An application on the PC (client) needs to initiate a communication session with the SimpleDAQ and sends a request(s). When the SimpleDAQ receives an incoming request, it performs the requested task(s) and transmits the results back to the client application (Figure 3). Requests and responses are in the form of byte messages (packets). An example of a request/response message is presented in Table 3.

**Figure 4.**
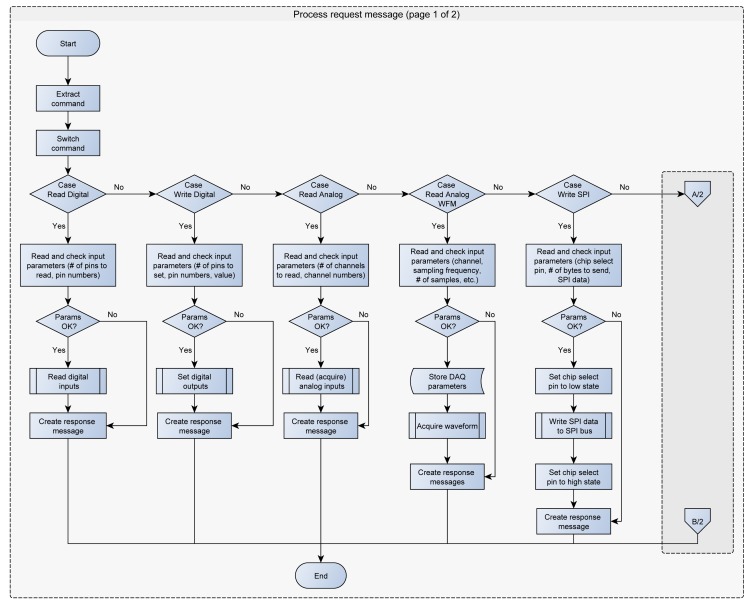
SimpleDAQ firmware flowchart: processing request message.

The maximum message size for the USB HID class is 64 bytes. All the request messages are smaller than 64 bytes, whilst the response messages can be larger. In this case the firmware splits the response message into several 64 bytes messages. The current version of SimpleDAQ firmware supports the following requests from the client: (1) read the state of one or more digital inputs, (2) set/reset one or more digital outputs, (3) acquire single value from one or more analogue inputs, (4) acquire waveform from analogue input, (5) configure the trigger parameters, (6) send (forward) the retrieved data to SPI bus, (7) configure the PWM frequency on PWM output, (8) configure the duty cycle on PWM output (9) reset SimpleDAQ and (10) configure SimpleDAQ pins according to the input configuration data. The flowchart of the request message processing procedure is presented in Figure 4.

More than half of the SimpleDAQ pins are flexible which means that the functionalities of these pins can be changed without microcontroller reprogramming. For example, an individual pin can be configured as digital input, digital output, analogue input, PWM output or one of three SPI lines. The pin configuration is achieved using the SimpleDAQ configuration utility (as described in the next section). This utility creates the configuration data and stores it within the microcontroller flash memory. At the microcontroller power-up (at the booting stage) the SimpleDAQ configures its pins regarding the settings stored within the flash memory.

#### 3.1.1. Digital Inputs/Outputs

All the SimpleDAQ flexible pins can be configured as digital input or digital output pins. If a pin is configured as digital output, an initial value (0 or 1) can be additionally defined.

#### 3.1.2. Analogue Inputs

**Figure 5.**
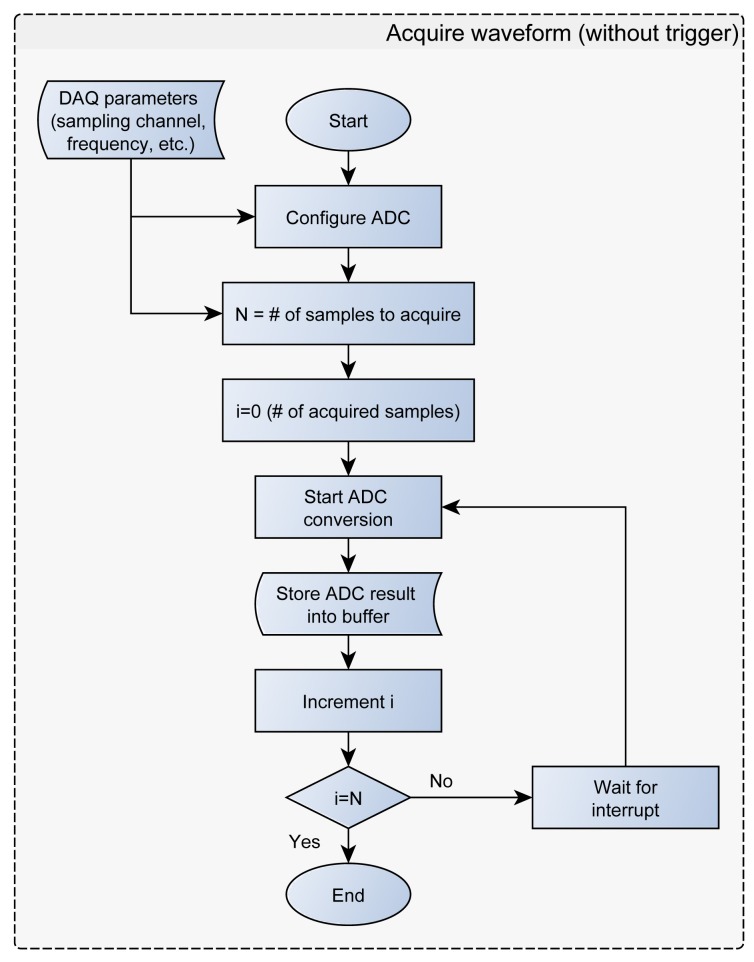
SimpleDAQ firmware flowchart: Waveform acquisition process.

The SimpleDAQ chip has up to 10 analogue inputs connected to a 12-bit ADC. Analogue inputs can be acquired as a single sample or finite samples (waveform). The latter option refers to the acquisition using predetermined sampling frequency and a predetermined number of data samples. In the finite sampling mode the samples are initially stored within the SimpleDAQ buffer. Once the specified number of samples has been acquired, the acquisition stops and the sampled data are transferred from the microcontroller buffer to the client application on the PC via USB (Figure 5). SimpleDAQ has the following limitations in the finite sampling mode: (1) the maximum sampling frequency is 100 kHz and (2) the maximum number of samples in the buffer is limited to 1024. These limitations are the consequence of microcontroller RAM size and the performance of the internal ADC. The ADC includes a self-calibration feature, which compensates for any offset generated within the module. The calibration process is automated and is initiated after each device reset. The calibration routine performs an offset calibration and stores the results internally. After that, all subsequent readings are automatically compensated.

##### Dynamic Performance of an ADC

In addition to the properties of the used ADC given in the microcontroller data sheet [24] the effective number of bits (ENOB) were measured in accordance with IEEE standards [26]. The ENOB is a way of quantifying the dynamic performance of an analogue-to-digital conversion [27]. For an input sine wave of specified frequency and amplitude, the ENOB is the number of bits of an ideal ADC for which the root-mean-square (rms) quantization error is equal to the rms noise and distortion of the ADC under test. The ENOB can be calculated from the signal-to-noise-and-distortion ratio, as expressed in dB (SINAD_dB_):

(2) $$\text{ENOB}=\frac{{\text{SINAD}}_{\text{dB}}-1.76+20\cdot \log\left(\frac{\text{FSR}}{A_{pp}}\right)}{6.02}$$

where FSR is the specified full-scale range of the ADC and *A_pp_* is the peak-to-peak amplitude of the applied sine wave.

In order to measure the ENOB a sine wave test signal with peak-to-peak amplitude 3 V and DC offset 1.65 V was applied to the ADC input of the SimpleDAQ module from the Tektronix AFG 3021B arbitrary function generator. The module was supplied from the PC via the USB port. The sine wave was then acquired using a SimpleDAQ LabVIEW driver, and the SINAD_dB_ was calculated using a LabVIEW built-in analysis function. Coherent sampling and uniform distribution of the samples in this phase were achieved by setting the input frequency according to [26]:

(3) $$f_{i}=\frac{J}{M}f_{s}$$

where:
*f_i_*Input signal frequency*f_s_*Sampling frequency*M*Number of samples in the test sequence*J*Integer number of input signal periods within the set sequence which is relatively prime to the *M*

**Figure 6.**
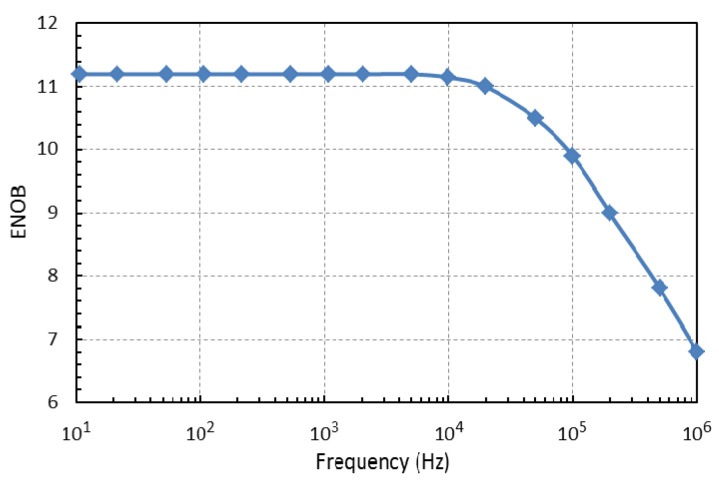
ENOB results for SimpleDAQ module.

The highest sampling frequency was 100 kHz and the number of samples was limited by the microcontroller’s memory to 1024. The test results for different frequencies between 10 Hz and 1 MHz are given in Figure 6. Note that the input frequencies above the Nyquist frequency *f_s_*/2 are aliased and that slightly better results could be achieved with low-pass and band-pass filtering of the input signal.

#### 3.1.3. Serial Peripheral Interface

SimpleDAQ also contains the serial peripheral interface (SPI) bus, through which it is possible to send data to other peripheral devices that support SPI communication. A SimpleDAQ chip has the role of master that can send the data to the various SPI devices (slaves). The master determines using a chip select pin as to which slave the data should be transmitted. The pin number of the desired chip select pin is retrieved from the request message form of the client application. This pin must be configured (using the SimpleDAQ Configuration Utility) as a digital output pin, and must have an initial value of 1. When the microcontroller retrieves the request for sending (forwarding) the data to SPI bus (Figure 4), it firstly extracts the chip select pin number from the input message and then initiates the SPI communication by turning the chip select pin to a low state. The microcontroller then forwards the received message from the USB to the SPI byte by byte. When it has finished sending the data, it turns the chip select pin back to high state and disables the SPI communication.

#### 3.1.4. PWM Outputs

The SimpleDAQ chip also has up to four PWM outputs. The firmware enables generation of PWM signals with a frequency of between 2.9 kHz and 100 kHz and a duty cycle of between 0 and 100%.

#### 3.1.5. Bootloader

**Figure 7.**
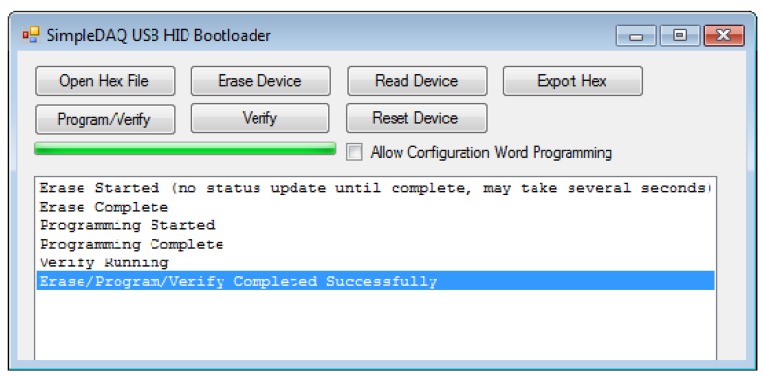
SimpleDAQ USB HID Bootloader PC application.

SimpleDAQ firmware includes a bootloader that enables upgrading of the firmware without the need for an external programmer or debugger. The bootloader code starts executing on device reset. If the bootloader condition is met (bootloader button pressed on the SimpleDAQ module), then the bootloader forces itself into the firmware upgrade mode (Figure 3). Otherwise, the firmware application code starts executing. In firmware upgrade mode the bootloader communicates with the PC host application (Figure 7) that is used to perform erase and programming operations. Bootloader waits for new data (new firmware) and loads it into the remaining part of the flash memory.

### 3.2. Configuration Utility

**Figure 8.**
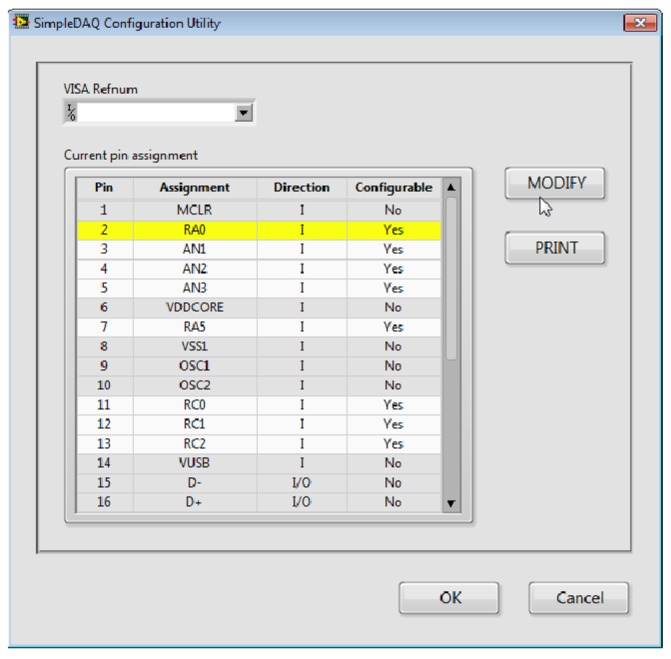
SimpleDAQ configuration utility.

**Figure 9.**
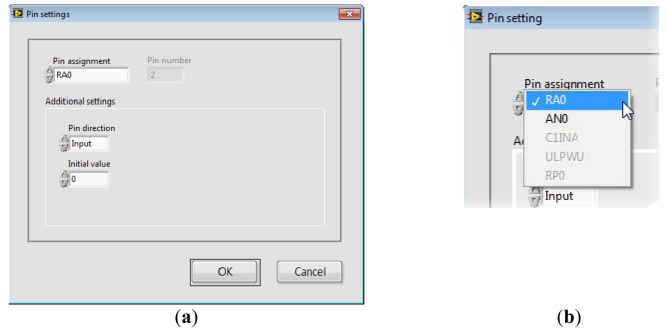
(**a**) Pin settings window (**b**) Pin assignment menu.

SimpleDAQ Configuration Utility (Figure 8) is a PC program that enables changing the functionalities of microcontroller pins without reprogramming. When using this utility, the microcontroller’s peripheral can be easily tailored to the needs of a customised application. In total, 18 out of 28 pins are configurable. The configuration utility (Figure 8) contains the *Current pin assignment* table with a list of all the SimpleDAQ pins. Modification of an individual pin can be achieved by selecting the desired pin within the *Current pin assignment* table and pressing the *MODIFY* button. In the *Pin Settings* window that appears (Figure 9), the user can select one of the pre-set options for the selected pin. Pin labels are taken from the microcontroller’s datasheet [24], as briefly explained in Table 4. By selecting an appropriate option (in *Pin Assignment* dropdown menu), additional options may appear that are specific to the selected option. For example, if an option RA0 (digital input or output) is selected the *Pin direction* and *Initial value* appear under the *Additional settings* section. When using the first option, the user can select the pin direction (input or output), whilst the second option enables the setting of the pin’s initial value (0 or 1), when the pin is configured as digital output.

The configuration utility creates the configuration data stream and sends it to the SimpleDAQ chip via USB. The SimpleDAQ firmware configures the pins according to the received configuration stream and also stores the retrieved configuration within the flash memory. At each SimpleDAQ reset, the firmware configures its pins regarding the settings stored in the flash memory.

### 3.3. LabVIEW Driver

**Figure 10.**
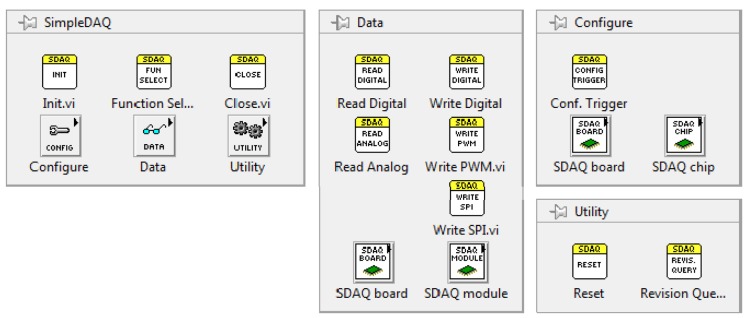
SimpleDAQ palette in LabVIEW.

A complete LabVIEW driver for the SimpleDAQ chip/module was developed. This driver enables easy access to the SimpleDAQ I/O peripheral using LabVIEW application on a PC. After the driver’s installation, all the SimpleDAQ-related virtual instruments (VI’s) appear on the SimpleDAQ palette, Figure 10. The short meanings of the more important SimpleDAQ VI’s are presented in Table 5.

#### Example of Using the LabVIEW Driver

**Figure 11.**
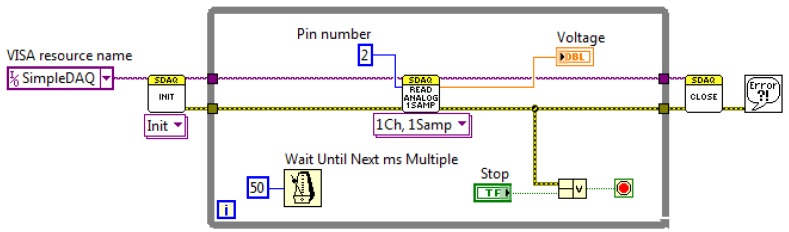
Acquisition of single sample using a software clock.

SimpleDAQ enables the acquisitions of analogue input signals using software or hardware clocks. An algorithm for the first option is shown in Figure 11. Functions *Init* and *Close* are used for establishing and closing USB connection with the SimpleDAQ device. At each While loop iteration one sampled value is retrieved using *Read Analog 1Ch, 1Samp* function. The software clock is defined using *Wait Until Next ms Multiple* timer function inside the While loop, Figure 11. In the presented algorithm, an analogue signal that is connected to pin 2 of the SimpleDAQ device is acquired every 50 ms.

**Figure 12.**
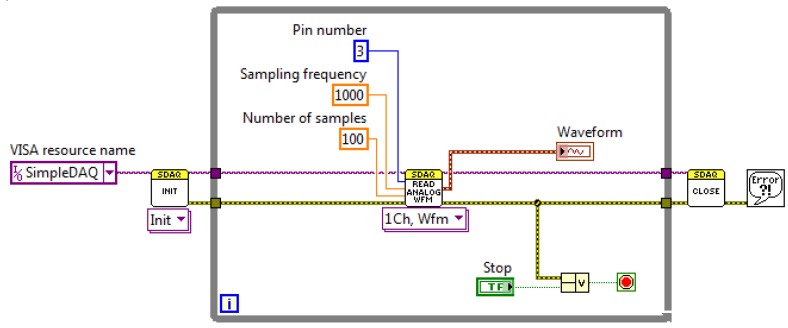
Acquisition of waveform using a hardware clock.

Acquisition of a finite number of samples using the hardware clock on a SimpleDAQ device is shown in Figure 12. The use of a hardware clock allows faster and more accurate signal acquisition. In this acquisition mode, the sampled data are initially stored within the buffer on the SimpleDAQ device. At the end of the acquisition process, the acquired data is transmitted to the PC application. Acquisition using a hardware clock is achieved using the *Read Analog Wfm* function in the LabVIEW algorithm (Figure 12). This function requires the following input parameters: (1) analogue input channel (chip pin number), (2) sampling frequency in Hz and (3) the desired number of samples.

## 4. An Example of an Application Board

A SimpleDAQ chip and module can be used on the breadboard or in combination with a custom-developed application board. Signals from the sensors often require signal conditioning such as amplification and filtering. This signal conditioning could be achieved using a custom application board. In addition to signal conditioning circuitry, an application board can also contain other external components, such as SPI chips.

**Figure 13.**
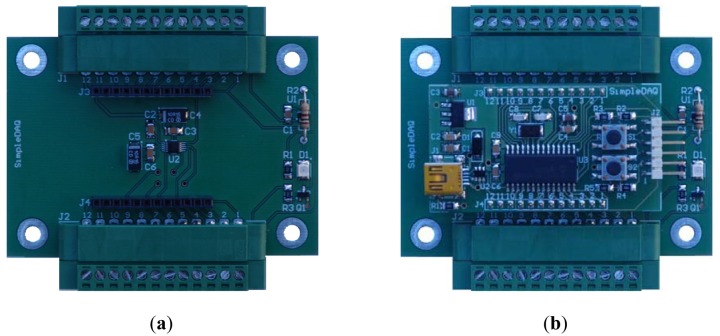
(**a**) An application board (**b**) An application board with an attached SimpleDAQ module.

An example of a custom application board is shown in Figure 13a. This board contains a low-power, programmable waveform generator [28] that is capable of producing sine, triangular, and square wave outputs. Waveform generation is required in various types of sensing and actuation applications. This chip has two 28-bit frequency registers and two 12-bit phase registers the values of which define the frequency and phase of an output waveform. The output frequency can be set from 1 Hz and to 1 MHz. The generator is connected to the SimpleDAQ module using a 3-wire SPI interface. In addition, this application board contains a screw terminal, LED indicator, analogue temperature sensor, and a heating element (resistor) that is placed above the sensor. The temperature sensor is the MCP9701A [29] from Microchip Technology. This sensor can accurately measure temperature from ‐40 °C to +125 °C. The output of the MCP9701A is calibrated to a slope of 19.3 mV/°C and has a DC offset of 400 mV. The offset allows measurements of the negative temperatures without the need for a negative supply.

(4) $$V_{out}=c_{T}\cdot T_{A}-V_{0{}^{\circ}C}$$

where *T_A_* is the ambient temperature, *V_out_* is the sensor output voltage, *V*_0°_*_C_* is the sensor output voltage at 0 °C (*V*_0°_*_C_* = 400 mV) and *c_T_* is the temperature coefficient (c*_T_* = 19.3 mV/°C)

**Figure 14.**
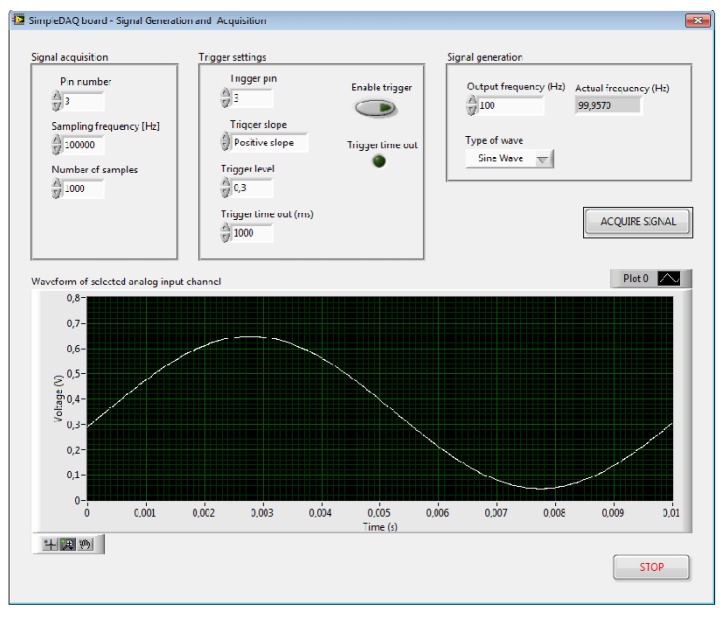
Application front panel.

**Figure 15.**
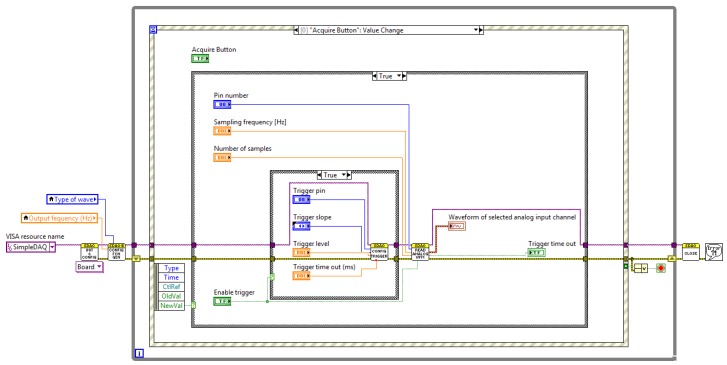
Application block diagram.

The output of the signal generator is connected to pin 3 of the SimpleDAQ module and the output of the temperature sensor to pin 1, respectively. This application board was developed especially for educational processes. A SimpleDAQ module in combination with this application board (Figure 13b) represents a simple and low-cost platform for signal generation, signal acquisition, and also for simple temperature control. Figures 14 and 15 present the LabVIEW application (front panel and block diagram) that enables signal generation and acquisition using this platform. The output of the signal generator is set at 100 Hz (sine wave), whilst an acquisition of this signal is performed using a hardware timer having a sampling rate of 100 kS/s.

## 5. Conclusions

This paper presented a flexible, microcontroller-based DAQ device. The presented hardware and software solutions enable easy realisations of custom data acquisition and signal processing algorithms, using microcontroller I/O peripheral and LabVIEW development environment. This approach eliminates tedious and time-consuming low-level microcontroller programming and enables developers to focus their energy onto custom analogue front-end development (if necessary) and data analysis algorithms’ developments. The presented solution is also highly appropriate for other technical fields (such as chemistry or mechanical engineering) where most technicians are not proficient in embedded programming but usually have a good knowledge of the LabVIEW programming environment. Unlike many related solutions where a microcontroller-based DAQ has been developed for a specific application, this solution is configurable and can be utilised for different customised applications.

In comparison to the commercially-available DAQ devices (Table 1) this solution has some advantages and disadvantages. Commercial products include analogue front end with configurable analogue inputs range. In contrast, the presented solution is without analogue front end and consequently the input range for I/O pins is limited to the microcontroller voltage level (between 0 V and 3.3 V). If the users have to use different input voltage ranges they have to develop their own application board with appropriate voltage level shifters. However, the possibility of using a custom-developed analogue front-end that is specific to the used sensors could actually be an advantage in some applications. DAQ applications vary according to the required I/O peripheral. For example, some applications require more analogue inputs whilst others more digital I/O’s. From this point of view SimpleDAQ is more flexible because the I/O peripheral can be changed, whilst the I/O peripheral of commercial available DAQs is fixed (for example, DAQ contains 8 AI, 2 AO, 8 DI 8 DO). In regard to the ADC sampling rate, SimpleDAQ is one of the best but it has a limited on-chip buffer. SimpleDAQ supports only single-ended analogue inputs whilst some commercial DAQs also support differential ones. SimpleDAQ does not contain any analogue output as the used microcontroller does not include a digital-to-analogue converter (DAC). However, analogue outputs can be realized using external SPI DAC chip on a custom application board. SimpleDAQ also includes some peripheral, which could not be found on commercial devices, such as SPI bus and PWMs. The SPI bus in particular is very usefully hence the variety of different SPI chips could be easily connected to the SimpleDAQ device.

The presented solution has many potential uses. It is relatively powerful, inexpensive, and flexible. The presented DAQ could also be used as a platform for custom-smart sensor development. When using the presented solution the prototype of a sensor application can be developed without microcontroller programming. Data acquisition, data analysis and decision-making algorithms can be developed on the PC. Once the developed algorithms have been proven and provide satisfactory results, they can be forwarded to a software developer group that writes optimised codes for this microcontroller.

To date this solution has been successfully tested on the Windows operating system. However, most operating systems (Windows, Linux, Android, *etc.*) natively support the USB HID class. In our future work, we will also develop example applications for other operating systems and programming environments (Java, Visual Basic, MATLAB, Delphi, *etc.*).

## Figures and Tables

**Table 1.** Off-the-shelf USB DAQ devices.

Company	Model	Key Features
Advantech	USB-4702-AE [19]	12-bit, 10 kS/s, 8 AI, 2 AO, 8 DI, 8 DO, 1 C/T
Data Translation	DT9812-10V [20]	12-bit, 50 kS/s, 8 AI, 2 AO, 16 DIO, 1 C/T
National Instruments	USB 6008 [21]	12-bit, 10 kS/s, 8 AI, 2 AO, 12 DIO, 1 C/T
LabJack	U12 [22]	12-bit, 8 kS/s, 8 AI, 20 DIO, 1 C/T
Measurement Computing	USB-1208FS-Plus [23]	12-bit, 50 kS/s, 8 AI, 16 DIO

Notes: AI – analogue input; AO – analogue output; DI – digital input; DO – digital output; DIO – digital input/output; kS/s – kilo samples per second; C/T – counter/timer.

**Table 2.** Microchip PIC18F27J53 specifications.

Parameter Name	Value
Program Memory Type	Flash
Program Memory	128 KB
RAM Bytes	3.800
CPU Speed	12 MIPS
Digital Communication Peripherals	2-A/E/USART, 2-MSSP(SPI/I2C)
Capture/Compare/PWM Peripherals	7 CCP, 3 ECCP
Timers	4 × 8-bit, 4 × 16-bit
A/D converter	10 ch, 12-bit
USB (ch, speed, compliance)	1, Full Speed, USB 2.0
Temperature Range	−40 to 85 °C
Operating Voltage Range	2 to 3.6 V
Pin Count	28
Package Type	SPDIP

**Table 3.** An example of a request/response message (acquire waveform from analogue input).

Message Type	Byte Number	Byte Meaning
Request message	0	Command (CMD_READ_ANALOG_WFM)
	1	Sampling channel (pin number)
	2	Reference (0: internal, 1: external)
	3	Trigger (0: disable, 1: enable)
	4	Sampling frequency (1. byte)
	5	Sampling frequency (2. byte)
	6	Number of samples (1. byte)
	7	Number of samples (2. byte)
	8	Trigger timeout in ms (1. byte)
	9	Trigger timeout in ms (2. byte)
Response message	0	Command (CMD_READ_ANALOG_WFM)
	1 to (2 · *Number of samples*)	Acquired data

**Table 4.** Pin assignment options in current version of firmware.

Pin Number	Pin Assignment Options	Assignment Meaning
2	RA0 | AN0	Digital I/O | Analogue input
3	RA1 | AN1	Digital I/O | Analogue input
4	RA2 | AN2	Digital I/O | Analogue input
5	RA3 | AN3	Digital I/O | Analogue input
7	RA5 | AN4	Digital I/O | Analogue input
11	RC0	Digital I/O
12	RC1	Digital I/O
13	RC2 | AN11	Digital I/O | Analogue input
17	RC6	Digital I/O
18	RC7 | SDO1	Digital I/O | Serial peripheral interface (SPI) data out
21	RB0 | AN12	Digital I/O | Analogue input
22	RB1 | AN10	Digital I/O | Analogue input
23	RB2 | AN8	Digital I/O | Analogue input
24	RB3 | AN9	Digital I/O | Analogue input
25	RB4 | CCP4 | SCK1	Digital I/O | PWM | SPI clock
26	RB5 | CCP5 | SDI1	Digital I/O | PWM | SPI data in
27	RB6 | CCP6	Digital I/O | PWM
28	RB7 | CCP7	Digital I/O | PWM

**Table 5.** The meanings of the more important SimpleDAQ virtual instruments.

Icon	Name	Palette	Meaning
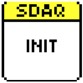	Init.vi		Function establishes USB connection between the SimpleDAQ chip/module and the PC.
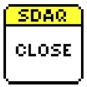	Close.vi		Function closes the USB connection between the SimpleDAQ chip/module and the PC.
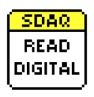	Read Digital.vi	Data	Polymorphic VI obtains the state of one or more digital input line(s).
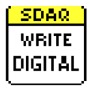	Write Digital.vi	Data	Polymorphic VI sets the state of one or more digital output line(s).
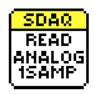	Read Analog.vi	Data	Polymorphic VI: Acquires one sampled value of the signal connected to the selected analogue input. Acquires the signal connected to the selected analogue input.
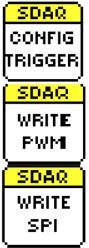	Configure Trigger.vi	Configure	VI sets the trigger settings.
	Write PWM.vi	Data	Polymorphic VI sets PWM frequency and PWM duty cycle.
	Write SPI.vi	Data	VI sends the input message to the SimpleDAQ SPI bus.

**Table 6.** Key components of an application board.

Component	Label
Programmable signal generator (AD9833)	U2
Temperature sensor (MCP9701A)	U1
Heating element (resistor)	R2
LED	D1
Screw terminals	J1 and J2
Sockets for SimpleDAQ module	J3 and J4
